# Supplementary material for: All-at-once RNA folding with 3D motif prediction framed by evolutionary information
Source: Nat Methods. 2025 Oct 3;22(10):2094–106. doi: 10.1038/s41592-025-02833-w (PMC12510878; doi:10.1038/s41592-025-02833-w)
Supplement: Supplementary file 3 — Source code to run CaCoFold-R3D. Data and results. [file 41592_2025_2833_MOESM3_ESM.gz › supplemental_material/Figures/Figure_S2.pdf]

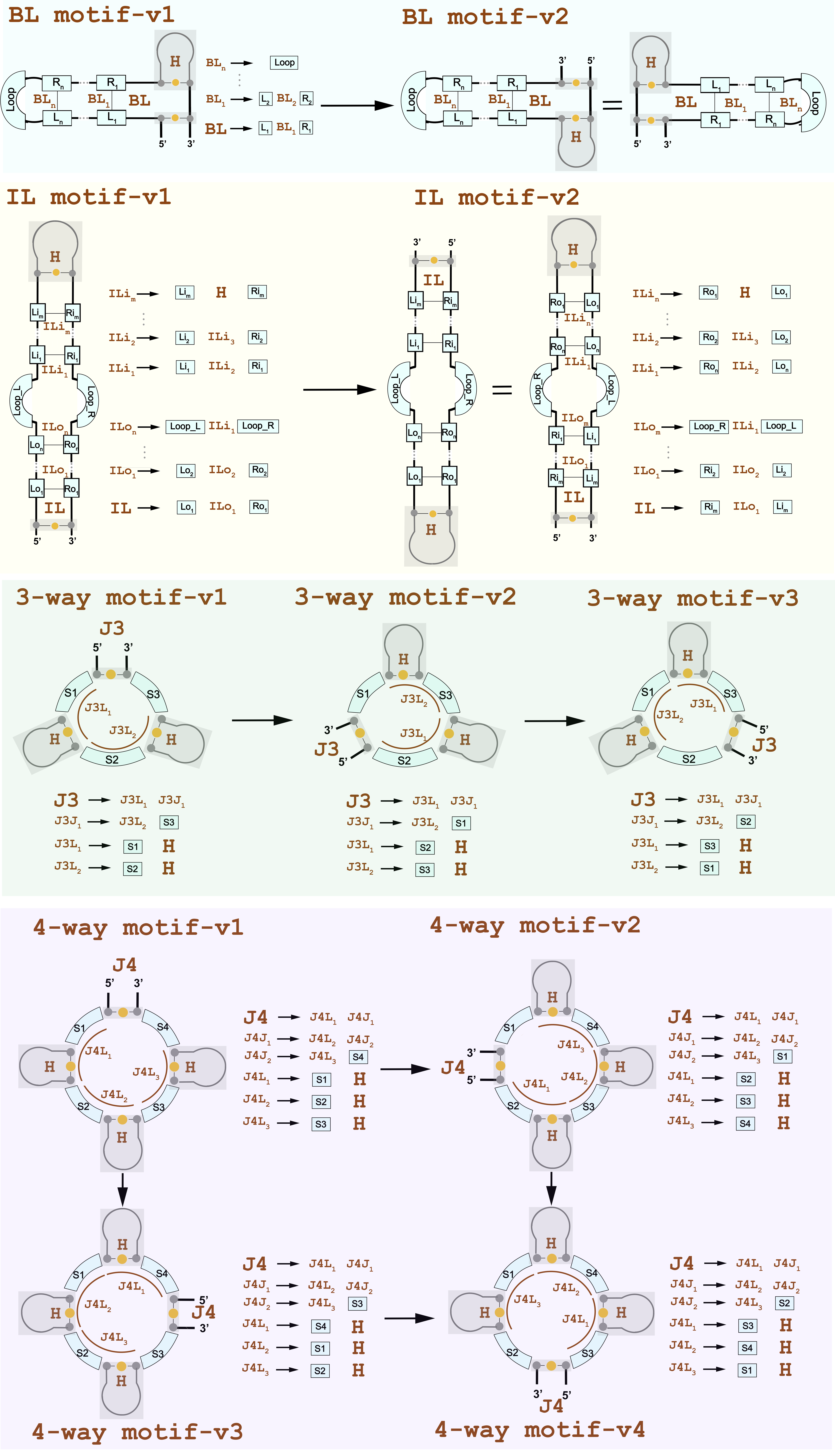

BL motif-v1
BL_n → Loop
BL_1 → L_2 BL_2 R_2
BL → L_1 BL_1 R_1
BL motif-v2
IL motif-v1
ILi_m → Li_m H Ri_m
ILi_2 → Li_2 ILi_3 Ri_2
ILi_1 → Li_1 ILi_2 Ri_1
ILo_n → Loop_L ILi_1 Loop_R
ILo_1 → Lo_2 ILo_2 Ro_2
IL → Lo_1 ILo_1 Ro_1
IL motif-v2
ILi_n → Ro_1 H Lo_1
ILi_2 → Ro_2 ILi_3 Lo_2
ILi_1 → Ro_n ILi_2 Lo_n
ILo_m → Loop_R ILi_1 Loop_L
ILo_1 → Ri_2 ILo_2 Li_2
IL → Ri_m ILo_1 Li_m
3-way motif-v1
J3 → $J3L_1$ $J3J_1$
$J3J_1$ → $J3L_2$ S3
$J3L_1$ → S1 H
$J3L_2$ → S2 H
3-way motif-v2
J3 → $J3L_1$ $J3J_1$
$J3J_1$ → $J3L_2$ S1
$J3L_1$ → S2 H
$J3L_2$ → S3 H
3-way motif-v3
J3 → $J3L_1$ $J3J_1$
$J3J_1$ → $J3L_2$ S2
$J3L_1$ → S3 H
$J3L_2$ → S1 H
4-way motif-v1
J4 → $J4L_1$ $J4J_1$
$J4J_1$ → $J4L_2$ $J4J_2$
$J4J_2$ → $J4L_3$ S4
$J4L_1$ → S1 H
$J4L_2$ → S2 H
$J4L_3$ → S3 H
4-way motif-v2
J4 → $J4L_1$ $J4J_1$
$J4J_1$ → $J4L_2$ $J4J_2$
$J4J_2$ → $J4L_3$ S1
$J4L_1$ → S2 H
$J4L_2$ → S3 H
$J4L_3$ → S4 H
4-way motif-v3
J4 → $J4L_1$ $J4J_1$
$J4J_1$ → $J4L_2$ $J4J_2$
$J4J_2$ → $J4L_3$ S3
$J4L_1$ → S4 H
$J4L_2$ → S1 H
$J4L_3$ → S2 H
4-way motif-v4
J4 → $J4L_1$ $J4J_1$
$J4J_1$ → $J4L_2$ $J4J_2$
$J4J_2$ → $J4L_3$ S2
$J4L_1$ → S3 H
$J4L_2$ → S4 H
$J4L_3$ → S1 H
